# The CpG Island Encompassing the Promoter and First Exon of Human *DNMT3L* Gene Is a *PcG/TrX* Response Element (PRE)

**DOI:** 10.1371/journal.pone.0093561

**Published:** 2014-04-17

**Authors:** Amitava Basu, Vasanthi Dasari, Rakesh K. Mishra, Sanjeev Khosla

**Affiliations:** 1 Centre for DNA Fingerprinting and Diagnostics (CDFD), Nampally, Hyderabad, India; 2 Centre for Cellular and Molecular Biology (CCMB), Council of Scientific and Industrial Research (CSIR), Hyderabad, India; CNRS, France

## Abstract

*DNMT3L*, a member of DNA methyltransferases family, is present only in mammals. As it provides specificity to the action of de novo methyltransferases, *DNMT3A* and *DNMT3B* and interacts with histone H3, *DNMT3L* has been invoked as the molecule that can read the histone code and translate it into DNA methylation. It plays an important role in the initiation of genomic imprints during gametogenesis and in nuclear reprogramming. With important functions attributed to it, it is imperative that the *DNMT3L* expression is tightly controlled. Previously, we had identified a CpG island within the human *DNMT3L* promoter and first exon that showed loss of DNA methylation in cancer samples. Here we show that this Differentially Methylated CpG island within *DNMT3L* (DNMT3L DMC) acts to repress transcription, is a Polycomb/Trithorax Response Element (PRE) and interacts with both PRC1 and PRC2 Polycomb repressive complexes. In addition, it adopts inactive chromatin conformation and is associated with other inactive chromatin-specific proteins like SUV39H1 and HP1. The presence of DNMT3L DMC also influences the adjacent promoter to adopt repressive histone post-translational modifications. Due to its association with multiple layers of repressive epigenetic modifications, we believe that PRE within the DNMT3L DMC is responsible for the tight regulation of *DNMT3L* expression and the aberrant epigenetic modifications of this region leading to *DNMT3L* overexpression could be the reason of nuclear programming during carcinogenesis.

## Introduction

In a eukaryotic cell, the presence of cis-regulatory elements ensures expression of genes at an appropriate level and in appropriate cells. Cis-regulatory elements not only include transcription factor binding motifs within the promoters but also DNA sequences located several kilobases away from the promoter that can positively or negatively influence the transcription rate of a gene [1]. Except for certain genes that are involved in housekeeping functions most genes are expressed in a tissue-specific manner. This tissue-specific regulation of genes is in turn achieved by interplay of the various cis-regulatory elements and their associated trans-acting factors. The importance of these regions in gene function can also be gauged by the fact that many of the disease causing mutations have been mapped to these cis-regulatory elements [2]. A well-studied example of cis-regulatory elements is the Polycomb/Trithorax Response Element (PRE). It was first identified in Drosophila but is present in most eukaryotic organisms and controls gene expression by recruiting Polycomb and Trithorax groups of regulatory proteins [3]–[6].

*DNMT3L* is a member of the Dnmt3 family of de novo DNA methyltransferases that includes *DNMT3A* and *DNMT3B*. DNMT3L lacks the catalytic domain and cannot methylate DNA on its own [7]. But it can influence DNA methylation by a non-specific mechanism through its interaction with DNMT3A and DNMT3B [8]. It also interacts with histone H3 at lysine 4. This interaction was found to be specific to the unmethylated form of lysine 4, indicating that DNMT3L could be the epigenetic effector that can read the histone code and postulated as a link between DNA methylation and histone modifications [9], [10]. Functionally, it has been shown to be involved specifically in setting up of DNA methylation during gametogenesis [11], [12]. Coincident with its function, *Dnmt3l* is expressed in mice during early embryogenesis and in the germ cells. It is also expressed at a very high level in ES cells [13]–[14]. In all other tissues, it is kept transcriptionally silent and this inactivity has been attributed to the epigenetic status of DNA sequences within and around the *DNMT3L* promoter [14], [15].

We had previously shown loss of DNA methylation for a CpG island spanning the human *DNMT3L* promoter/exon1 region (promoter for *DNMT3L* variant 2 and first exon in case of *DNMT3L* variant 1) for cervical and ocular cancer samples [16], [17]. Since there is a genome-wide nuclear reprogramming associated with carcinogenesis, the loss of DNA methylation observed in the CpG island around the *DNMT3L* promoter could either be coincidental to the process of carcinogenesis or has a role to play during carcinogenesis. In addition, the loss of DNA methylation at this CpG island could be indicative of a role for this region in the regulation of the *DNMT3L* expression. To examine this, we sought to analyse the functional role of this region in regulating transcription. In the present study, we have shown, by performing reporter gene assays in mammalian cell lines and Drosophila, that the DNA sequence present within the *DNMT3L* promoter/Exon1 acts to repress transcription. This region acts as a Polycomb/Trithorax Response element (PRE) and mediates repression by adopting inactive-chromatin-specific histone modifications through its interaction with Polycomb group of proteins.

## Materials and Methods

### Transient transfection assay in mammalian cell lines

**Figure 1:**
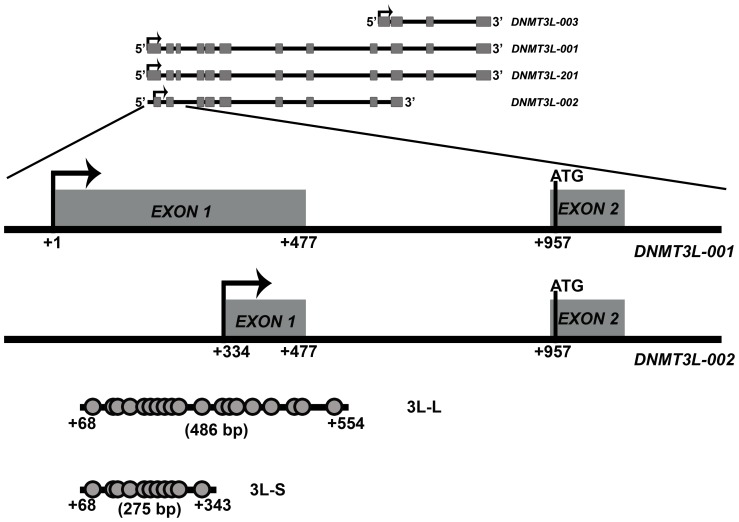
Graphical representation of the alternate Transcription Start Sites for the human *DNMT3L* gene. The human *DNMT3L* gene produces four transcripts (not drawn to scale) from three different Transcription start sites (TSS, raised arrows). Exons are shown as grey rectangles. Shown here in detail are two TSS which are responsible for the DNMT3L-001 & DNMT3L-201 (from +1) and DNMT3L-002 (+334) transcripts. Only DNMT3L-001 and 002 are shown in the representation. The most 5′ TSS has been taken as +1 position. Translation start site (shown as vertical line with ATG) is the same (at +952, in the second exon) for all these three *DNMT3L* transcripts. However, all the three differ in the length of the final DNMT3L protein. Protein made by DNMT3L-001 and DNMT3L-201 are 387 aa and 386 aa long respectively, DNMT3L-003 is 181 aa and DNMT3L-002 makes a 288 aa long protein. The fragments 3L-L and 3L-S from this region were analysed for their function by reporter gene assays in the present study. The approximate positions of the CpG dinucleotides within these fragments are shown with filled circles. All nucleotide positions mentioned are relative to the most 5′ TSS site.

The CMV promoter fragment was PCR amplified from pEGFPC3 vector (Clontech) and cloned between the *Eco*RI and *Bam*H I sites of the promoter-less pAcGFP1-1 vector (Clontech) to derive the pCMV-AcGFP1-1 plasmid. 3L-S and 3L-L regions from the *DNMT3L* locus (figure 1) were PCR amplified from the HeLa genomic DNA using the primers 3LF (5′-CCTGAGGGCCCCATCCTCTG-3′) and 3LSR (5′-AAGGATCCAGGCCCACCTGGGAC-3′) for 3L-S and 3LF and 3LLR (5′-CAGGGACCCCTGGGGATGGTCTTGGCC-3′) for 3L-L. The fragments were cloned in both orientations upstream of the CMV promoter in pCMV-AcGFP1-1 vector between *Xho*I and *Eco*RI. As a negative control we cloned a 1 kb region from human chromosome 1, which was previously shown to have no transcriptional potential upstream of the CMV promoter [18]. A 1.5 kb region from the H19 ICR, which has been shown to be a transcriptional repressor [19] was also cloned upstream of the CMV promoter (Figure 1). As a control for transfection efficiency we used the pG5luc vector (Promega) containing the luciferase reporter gene. Transfections were done in HEK293 cells using Lipofectamine 2000 (Invitrogen) in duplicates following the manufacturer's protocol.

### Expression analysis of the Reporter gene

48 hrs post transfection, cells were harvested and analysed for AcGFP and Kan/Neo^R^ transcript levels by Real-time PCR using the Mesa Green qPCR Mastermix plus (Eurogentec cat no 05-SY2X-03+WOU) in the ABI Prism SDS 7500 system with the following primer sets:

For *AcGFP*

AcGFPF 5′TCCTGGGCAATAAGATGGAG 3′

AcGFPR 5′ TGGGGGTATTCTGCTGGTAG 3′

For *Neo^R^*

Neo^R^F 5′ GGAGAGGGCTATTCGGCTATG 3′

Neo^R^R 5′ GACAGGTCGGTCTTGATAAA 3′

For *GAPDH*

GAPDHF 5′ TGCTGGCGCTGAGTACGTCG 3′

GAPDHR 5′ GGGTGGCAGTGATGGCATGG 3′

*GAPDH* was used as an internal control in Real-Time PCR.

To examine the levels of AcGFP protein, the respective constructs were co-transfected and Western blotting was performed as per established protocol [20]. The transfection efficiency was controlled by co-transfecting the Luciferase gene containing pG5luc vector. β-TUBULIN was used as loading control. GFP antibody was from Sigma and the β-TUBULIN antibody was from Abcam. The band intensities were determined by performing a densitometric scan using the Alpha Ease FC software (Alpha Innotech Corporation). Luciferase assay was done using the Luciferase Assay System Kit (Promega) following the manufacturers instruction and the light intensity was measured in the Varioskan Flash Multimode Reader (Thermo Scientific). The GFP protein level was calculated by normalizing the GFP/β-TUBULIN ratio for each construct with their respective Luciferase activity (Relative Light Unit at 0.1 sec per reading integration time). The final value was calculated as a percentage of GFP expression for each construct relative to the expression of GFP in the control CMV only construct (which was taken as 100%). Each experiment was performed thrice and was done in duplicates.

### Reporter gene assay in Drosophila

Both 3L-S and 3L-L, cloned between the two *LoxP* sites of the pLML vector were subcloned in the Drosophila vector, pCaSPeR, upstream of the reporter *miniwhite* gene that was under the control of *hsp70* promoter. The sequence as well as the orientation was confirmed by sequencing. The constructs for microinjection in Drosophila were purified by midi-prep kit (Qiagen). Microinjection was done in the Drosophila white eyed mutants *w^1118^* as per established protocols [21]. All the lines were balanced using the double balancer flies to establish independent balanced stocks. The 3L-L fragment was excised from various transgenic lines by crossing transgenic lines to the *Cre* recombinase-expressing Drosophila flies as described previously [22]. The transgenic lines as well as the respective flipped out lines were checked by PCR to ensure that 3L-L region had been successfully flipped out. The primers used for checking out the flipped out lines were

3L-LF-5′GCAGTATGCCGTTTACTGTGTG3′ and

3L-LR-5′CCCCGATCCCCCTAGAATCCCAAA 3′

To examine the localisation of the construct in the genome, inverse PCR was performed using the following primers:

Plac1- 5′ CACCCAAGGCTCTGCTCCCACAAT 3′

Pwht1- 5′ GTAACGCTAATCACTCCGAACAGGTCACA 3′

### Drosophila Eye pigmentation assay

20 heads from flies of similar age and sex were homogenised in 1∶1 mixture of Chloroform and 0.1% Ammonium hydroxide mix. The homogenate was centrifuged at low speed to pellet down the debris and absorbance of the supernatant was taken at 485 nm using Chloroform/Ammonium Hydroxide mixture as the blank [23]. The experiment was repeated thrice.

### Crosses with Drosophila mutants

To determine the epigenetic effectors that interact with the 3L-L region in Drosophila, we set up a series of crosses with the various Polycomb and Trithorax mutant flies. The transgenic lines were crossed to various mutant stocks that were in white eye background. The Trithorax mutants used in these crosses were: *Ash2^1^*, *Mor^1^*, *Brm^2^*, *Trl^R85^* and *Trx^1^* while *Pc^1^*, *esc^2^*, *Asx^XF53^*, *Psc^1^*, *Pcl^T1^*, *Scm^R5-13B^*, *Phd* and *Pho* were the Polycomb mutants used. Mutants for the epigenetic modulators that enhance or suppress position effect variegation including *Su(var)2-5^01^* and *Su(var)3-9^06^* were also used [24], [25].

### Chromatin immunoprecipitation

Isolation of chromatin from Drosophila larvae was done following established protocol (see Supplementary Information S1). To isolate chromatin for ChIP analysis from transfected cells, 1×10^6^ cells transfected cells were incubated with 1% formaldehyde at 37°C for 10 minutes. The reaction was quenched by incubation with 125 mM Glycine. The medium was removed and the cells were washed twice with PBS containing protease inhibitors. Cells were collected centrifuged at 300 g and resuspended in 400 µl of lysis buffer (1% SDS, 10 mM EDTA and 50 mM Tris pH 8). The lysate was sonicated at maximum power for 12.5 minutes with 30 sec off/on cycle to obtain DNA fragments between 200–1000 bp in a bioruptor™ (Diagenode).

For Chromatin Immunoprecipitation with antibodies against various histone modifications and other epigenetic effectors we performed the protocol described by antibody manufacturer (Millipore). Antibodies to H3K4me3, H3K9ac, H3K9me3 and H3K27me3 (Millipore) were used in our ChIP analyses. Rabbit IgG (Sigma) was used as a control. 1% of chromatin was taken as input and processed separately. The enrichment of the various proteins or Histone modification at the region of interest was evaluated by performing Real time PCR using Mesa Green qPCR Mastermix plus (Eurogentec) in the ABI Prism SDS 7500 system. To evaluate the efficiency of our ChIP assays, we performed Chromatin Immuno-precipitation for known regions in the human genome with the antibodies that we used in our assay (Figure S1, based on literature provided by Abcam, the manufacturer of the antibodies used). The primers used in our assay were

*In Drosophila*

For 3L-L region

ChIP3L-LF- TGGATCCTTCCAGCTCATTC and

ChIP3L-LR-GCATGGTCAGGGAGTAGGAA

For *hsp70* promoter

Hsp70F 5′ TGCAGTTGATTTACTTGGTTGC 3′

Hsp70R 5′ ATAGAGCGCTTCGTCTACGC 3′

*In mammalian transfection study*

For 3L-L region

pAcGFP3LLF 5′ TAGCGCTACCGGACTCAGAT 3′

pAcGFP3LLR 5′AAAGAATCTGGGCGTGTCC 3′

For CMV promoter

CMVF 5′ TCCGCGTTACATAACTTACGG 3′

CMVR 5′ GTCAATAGGGGGCGTTCTTG 3′

For GAPDH control oligos

GAPDH F 5′ TACTAGCGGTTTTACGGGCG 3′

GAPDH R 5′ TCGAACAGGAGGAGCAGAGAGCGA 3′

For SAT2 control oligo

SAT2 F 5′ CTGCAATCATCCAATGGTCG 3′

SAT2 R 5′ GATTCCATTCGGGTCCATTC 3′

For MyoD control oligo

MyoD F 5′ CCTCTTTCGGTCCCTCTTTC 3′

MyoD R 5′ TTCCAAACCTCTCCAACACC 3′

Each ChIP followed by Real-time PCR was performed thrice and done in duplicates.

### DNA Methylation analysis by bisulfite sequencing

Sodium bisulphite modification of DNA was performed using BisulFlash DNA modification kit (Epigentek) according to the manufacturer's protocol using 1 µg of genomic DNA. Modified DNA was collected in 20 µl of Elution Buffer. Methylation-specific PCR was set up using the following primers:

CMV promoter

BisCMVF 5′ ATTTGGTAGTATATTAAGTGTATTATATGT 3′

BisCMVFR 5′ AACTCTACTTATATAAACCTCCC 3′

3L-L region

Bis3LLF 5′ TTGAGTTATTTTTTGATTTTGTGGATAA 3′

Bis3LLR 3′ CAAAAATCCAAACCCACCTAAA 3′

Endogenous 3L-L region

Bis 3LL F 5′ TGTTTTTATAGTTTGGTGATTTTTGG 3′

Bis 3LL R 5′ CCCCTAAAAATAATCTTAACCAACC 3′

The PCR products obtained were cloned using pCR2.1-TOPO cloning kit (Invitrogen). For each region a minimum of 15 clones were sequenced to confirm the methylation pattern.

### Gene knock-down by siRNA

The siRNA knock-down of the polycomb proteins *EZH2* and *EED* and suppressor of varigation protein, *SUV39H1* was performed in HEK293 cells using the siRNA Smart Pools for these proteins (Thermo Scientific Dharmacon) according to the manufacturer's protocol. Expression of the endogenous *EZH2*, *EED* and *SUV39H1* was assayed by qRT-PCR. The downregulation of the individual genes in specific-siRNA transfected cells was quantitated in comparison to that observed for cells transfected with scrambled siRNA (from Thermo Scientific Dharmacon). The endogenous expression level of *DNMT3L* was measured in the knock-down and the untreated cells by qRT PCR thrice and in duplicates.

RT-PCR primers for the respective genes are as follows:

For Suv39H1

Suv39H1F – 5′GCGTATCCTCAAGCAGTTCC3′

Suv39H1R – 5′TCCAGGTCCACCTCATTCTC3′

For EZH2

EZH2F- 5′ATGCGACTGAGACAGCTCAA3′

EZH2R- 5′TGGGATGACTTGTGTTGGAA3′

For EED

EED F- 5′GAGAGGGAAGTGTCGACTGC3′

EED R- 5′CATTTTCCCTTTCCCCAACT3′

For CYCLOPHILIN B

CYCF- 5′CTTCCCCGATGAGAACTTCA3′

CYCR- 5′TTATCCCGGCTGTCTGTCTT3′

For endogenous *DNMT3L*

DNMT3L F- 5′CTCTCAAGCTCCGTTTCACC3′

DNMT3L R- 5′GTACAGGAAGAGGGCATCCA3′

## Results

### Functional analysis of the *DNMT3L* promoter/Exon 1 region

In our previous study, we had identified a CpG island spanning the promoter (DNMT3L-002, Ensembl Transcript ID ENST00000431166) and Exon 1 (DNMT3L-001, Ensembl Transcript ID ENST00000270172) of the human *DNMT3L* gene that showed loss of DNA methylation in different cancers [16], [17]. The loss of DNA methylation within this region was also shown to correlate with increased *DNMT3L* expression [15]. As this region showed differential DNA methylation profiles in normal and cancer samples we have referred to it as DNMT3L DMC (Diffferentially Methylated in Cancer) in this manuscript.

To examine whether this CpG island within the *DNMT3L* promoter/Exon1 exerts a cis-regulatory role, we performed reporter gene assays in mammalian cell lines and Drosophila.

### Transient transfection assay in cell lines

**Figure 2:**
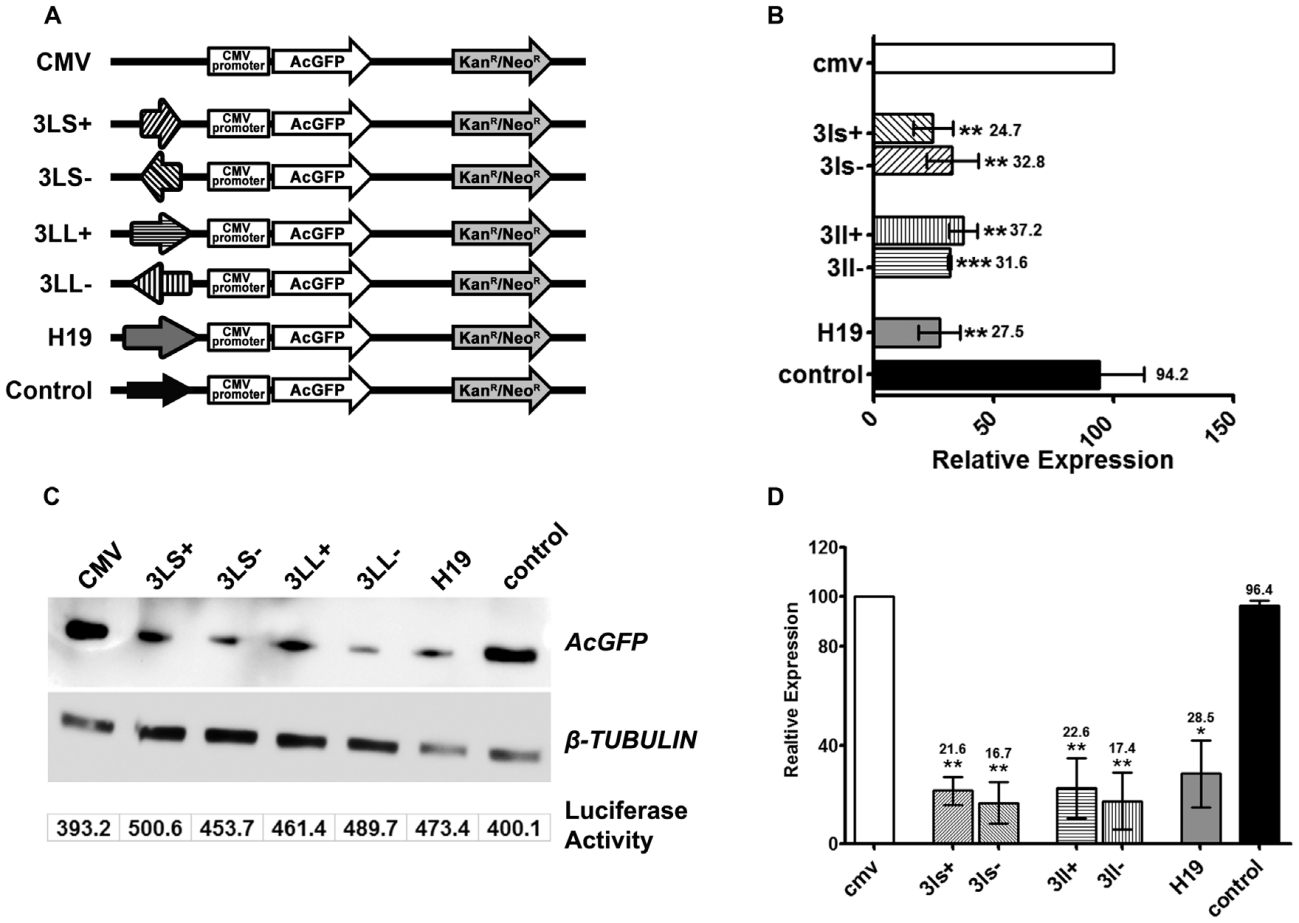
Functional analysis of DNMT3L DMC by reporter gene assay in transiently transfected mammalian cells. A. Graphical representation of the various constructs transfected into HEK293 cells. Shown for each construct is the reporter AcGFP gene, its CMV promoter, the selection Kan^R^/Neo^R^ marker, the various DNMT3L DMC fragments, the control Chr1 fragment and the H19 ICR inserted upstream of the CMV promoter. B. Relative transcriptional level of the reporter AcGFP gene in the various constructs. The transcriptional levels were measured by Real-Time PCR and calculated relative to the AcGFP expression in the control CMV only construct. *Kan* ^R^/*Neo* ^R^ expression level was used to control for transfection efficiency in the Real-Time PCR analysis. C. A representative Western Blot analysis showing AcGFP protein expression for HEK293 cells transfected with the various constructs mentioned in A. β-TUBULIN was used as a loading control. Luciferase activity for the pG5luc vector co-transfected with the above mentioned constructs is shown below the β-TUBULIN panel. D. Relative AcGFP protein expression levels for the cells transfected with the various constructs. Error bars represent Standard Deviation (S.D.). Asterisks indicate significant difference (Student's t test, * - p<0.05, ** - p<0.01, *** - p<0.001).

For functional analysis of the DNMT3L DMC, we cloned two overlapping fragments from this region into the pAcGFP-CMV vector (Figure 1, see Materials and Methods). The smaller region 3L-S contained the 11CpGs that were examined in our previous study [16]. The larger fragment 3L-L contained 19 CpG (including all the 11 present in 3L-S, Figure 1). Both the fragments were cloned upstream of the CMV promoter in both orientations. A 1 kb region from human chromosome 1, which was previously shown to have no effect on transcriptional potential of a promoter and a 1.5 kb region from the H19 ICR, which has been shown to be a transcriptional repressor were also examined in our assay (see Materials and Methods and Figure 2A, [18], [19]). The constructs were transfected into HEK293 cells and 48 hours after transfection, the GFP expression for the various constructs was examined either by Real-time RT-PCR or Western. *GAPDH* was used as control in each Real-Time RT-PCR experiment. To control for the transfection efficiency, ratio of *GFP* transcription and *Kan^R^/Neo^R^* expression (part of the same pAcGFP-CMV vector) was calculated. As can be seen in figure 2B, the presence of 3L-L and 3L-S in both orientations significantly decreased the expression level of GFP. The approximately 70% decrease in the *GFP* expression was comparable to that observed for the H19 ICR, a known transcriptional repressive element [19]. For Western analysis of GFP expression, transfection efficiency was controlled by co-transfecting pG5luc vector containing the Luciferase reporter gene. A representative blot is shown in figure 2C. Ratio of band intensity for GFP and β-TUBULIN proteins was calculated and the values were normalised for the transfection efficiency with the Luciferase activity. As was observed for real-time RT-PCR analysis, the presence of 3L-L and 3L-S in both orientations significantly decreased the expression level of GFP by more than 70% (figure 2D).

### Identification of the minimal region within the DNMT3L DMC that can repress transcription

**Figure 3:**
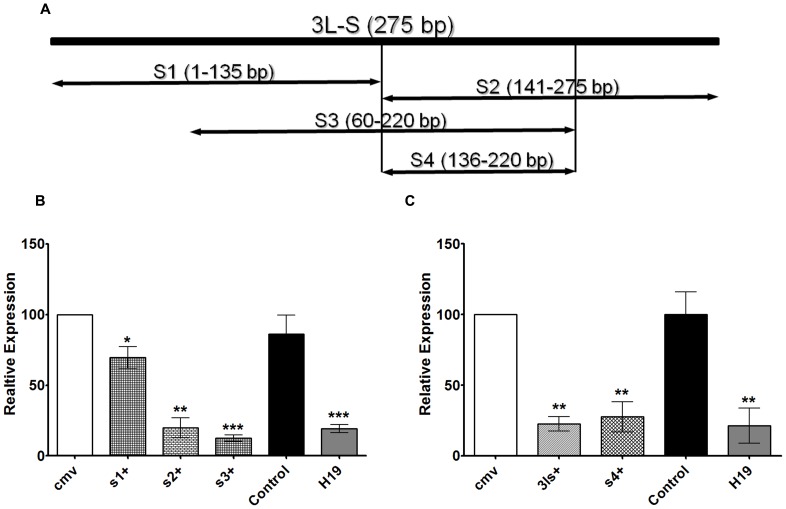
Identification of the minimal region within the DNMT3L DMC that can repress reporter gene transcription. A. Graphical representation of the smaller fragments of the 3L-S region which were cloned into the AcGFP reporter gene vector and transfected into HEK293 cells (see figure 2A for the details of the vector). Base-pair position of each fragment with respect to the 3L-S fragment is given within brackets. Vertical lines denote the minimal region that can repress reporter gene transcription. Subfragments of 3L-S have been named s1, s2, s3 and s4. B & C- Quantitative RT-PCR was performed to determine relative transcriptional level of the reporter AcGFP gene in the various constructs. The relative transcriptional levels were measured with respect to the AcGFP expression in the CMV only construct. See figure 2A for information on the Control and H19 constructs. Error bars represent Standard Deviation (S.D.). Asterisks indicate significant difference (Student's t test, * - p<0.05, *** - p<0.001, *** - p<0.005).

To identify the minimum region within the DNMT3L DMC that can repress transcription we cloned three overlapping sub-fragments of 3L-S (termed as s1, s2 and s3) in to the pAcGFP-CMV vector (figure 3A). The GFP expression for the various constructs was examined 48 hours after transfection into HEK293 cells by Real-time RT-PCR. The s2 and s3 constructs showed approximately 70% decrease but s1 construct showed only 20% decrease in GFP expression (figure 3B). Since s2 and s3 were overlapping fragments, it was decided to examine whether the overlap region (designated as s4) was enough to cause transcriptional repression. As can be seen in figure 3C, s4 construct also showed approximately 70% decrease in GFP expression.

### Transgene reporter assay in Drosophila

**Figure 4:**
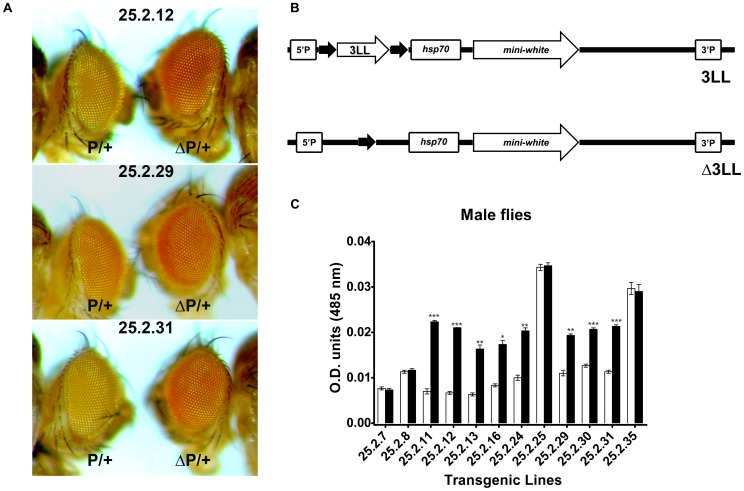
Functional analysis of DNMT3L DMC by reporter transgene assay in Drosophila. A. The three panels show comparison of eye color phenotype between representative DNMT3L DMC transgenic lines and their counterpart lines where the inserted 3L-L region had been flipped out using the surrounding *lox*p sites. P/+, heterozygous 3L-L transgenic lines; ΔP/+ their respective flipped-out counterparts. B. Graphical representation of the reporter gene construct pCaSPeR-3L-L and it's flipped out counterpart (Δ3LL). The construct contains the *mini-white* reporter gene under the control of *hsp70* promoter. The 3L-L region (shown as unfilled arrow) was inserted upstream of the *hsp70* promoter between the *loxP* sites (filled arrows). Only one *loxP* site remains in the flipped out counterpart. 5′P and 3′P refer to P element present 5′ and 3′ to the reporter gene. C. Comparison of eye color pigmentation between representative 3L-L transgenic lines and their flipped out counterparts. Numbers below the X-axis denote names of individual transgenic lines. White bars represent P/+ lines whereas black bars represent their ΔP/+ flipped out counterparts. Error bars represent Standard Deviation (S.D.). Asterisks indicate significant difference (Student's t test, * - p<0.05, ** - p<0.01, *** - p<0.001).

To examine the role of DNMT3L DMC in a *mini-white* reporter gene assay in Drosophila the 3L-L fragments flanked by the *lox*P site was cloned upstream of the *hsp*70 promoter in the Drosophila cloning vector pCaSPeR and injected into *w^1118^* Drosophila embryos (Figure 4B and Materials and Methods). It was decided to continue further with only the 3L-L Drosophila transgenic line as we had observed similar levels of transcriptional repression for both 3L-L and 3L-S in transient transfection assay in mammalian cells. Even after several rounds of injections, wherein we injected the 3L-L construct into around 1500 embryos, we obtained only one transgenic line for 3L-L. To take in consideration the effect for integration site on the expression of a transgene in Drosophila, we mobilised the 3L-L fragment from the only transgenic line which was obtained for 3L-L by crossing it to the flies carrying Δ2-3 transposase gene and expressing transposase endogenously. Upon, mobilisation, we obtained 12 independent heterozygous 3L-L transgenic lines (Table 1). The eye colour varied from very light yellow to deep red due to position effect. To check the effect of the 3L-L on the *mini-white* gene expression, we flipped-out the 3L-L region in these lines by crossing them to the *cre*-transgenic Drosophila line [22]. Eye colour comparison was done both visually under the microscope and by the eye pigmentation assay between the 12 transgenic lines and their respective flipped-out lines (figure 4A). For 8 out of the 12 3L-L transgenic lines, a statistical significant increase in eye colour pigmentation was observed in the flipped-out counterparts (figure 4C, Table 1) suggesting that the 3L-L region was inhibiting transcription of the *mini-white* reporter gene. We confirmed the genomic localisation of the 3L-L construct in these 8 transgenic lines by inverse PCR. Except for 25.2.12 and 25.2.30, all the transgenic lines had 3L-L construct at different loci in the genome (Table S1).

**Table 1:** Eye color phenotype of the various DNMT3L DMC transgenic lines and their flipped-out counterparts.

LINE	Eye color (3LL)	Eye color (Δ3LL)	Relative Pigmentation (%[Δ3LL/3LL])
**25.2.7**	Light Yellow	Light Yellow	96.05
**25.2.8**	Yellow	Yellow	102.65
**25.2.11**	Light Yellow	Orange	318.57
**25.2.12**	Light Yellow	Orange	333.33
**25.2.13**	Light Yellow	Light Orange	258.73
**25.2.16**	Light Yellow	Light Orange	208.43
**25.2.24**	Yellow	Orange	203
**25.2.25**	Red	Red	101.76
**25.2.29**	Yellow	Light Orange	175.45
**25.2.30**	Yellow	Light Orange	171.42
**25.2.31**	Yellow	Light Orange	188.49
**25.2.35**	Red	Red	105.07

The approximate eye color for each line is mentioned. Relative eye pigmentation was calculated as percentage change between each transgenic line and its flipped-out counterpart.

### Characterisation of interaction between DNMT3L DMC with Polycomb and Trithorax proteins

**Figure 5:**
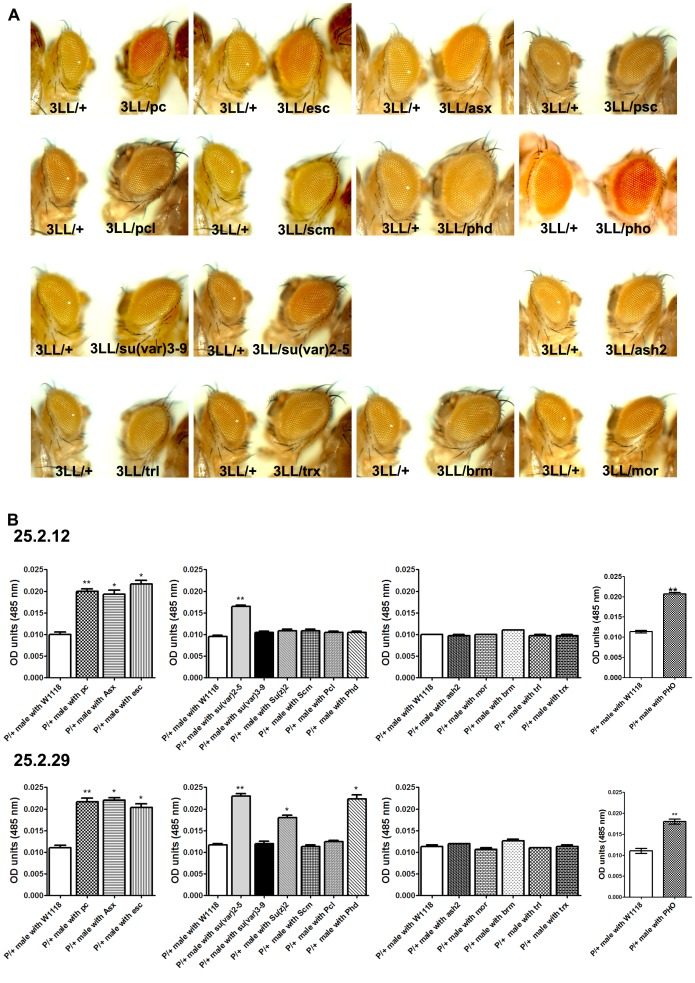
DNMT3L DMC interacts with Polycomb group of proteins. A. The 15 panels show comparison of eye color phenotype between representative DNMT3L DMC transgenic lines (25.2.12) and their counterpart lines after crossing with the respective Polyomb (*Pc^1^*, *esc^2^*, *Asx^XF53^*, *Psc′*, *Pcl^T1^*, *Scm^R5-13B^*, *Phd*, *Pho*), Trithorax (*Ash2^1^*, *Mor^1^*, *Bbrm^2^*, *Trl^R85^*, *Trx^1^*) and Supressor of Variegation (*Su(var)2-5^01^*, *Su(var)3-9^06^*) mutants. 3LL/+, heterozygous 3L-L transgenic lines; 3LL/−, their respective counterparts after crosses with the respective mutant (− is the name of the Polycomb, Trithorax or Suvar mutant). B. Comparison of eye color pigmentation between 3L-L transgenic lines (25.2.12 and 25.2.29) and their counterparts from crosses with the various mutant lines. Each bar represents eye color pigmentation for progeny from crosses of individual transgenic lines with a particular mutant, the details of which are provided below the X-axis. As the assays were done in batches, the eye pigmentation for the control 3L-L transgenic line was done for each batch and is shown as white bars (P/+ male with *W^1118^*) in the graphs. Error bars represent Standard Deviation (S.D.). Asterisks indicate significant difference (Student's t test, * - p<0.05, ** - p<0.01).

Polycomb and Trithorax group of proteins are known regulators of gene expression. The functions performed by the two groups are antagonistic. Polycomb group of proteins are involved in repressing gene expression and creating a highly condensed chromatin conformation whereas Trithorax group of proteins help in enhancing gene expression and keeping a gene in active state of chromatin organisation [4], [26]. Polycomb group (PcG) of proteins are known to bind to the Polycomb Response Elements (PRE) thereby preventing the binding of transcription factors to the DNA [27]. It is also believed that the PcG proteins bring about the deacetylation of the histones [28]. To test whether Polycomb or Trithorax group are involved in the observed transcriptional repression by the DNMT3L DMC, two of the eight 3L-L transgenic Drosophila lines (25.2.12 and 25.2.29) were crossed with the following mutant Drosophila lines: *Ash2^1^*, *Mor^1^*, *Brm^2^*, *Trl^R85^* and *Trx^1^* (Trithorax group); *Pc^1^*, *esc^2^*, *Asx^XF53^*, *Psc^1^*, *Pcl^T1^*, *Scm^R5-13B^*, *Phd and Pho* (Polycomb group). We also set up crosses with the histone methyltransferases, *Su(var)2-5^01^* and *Su(var)3-9^06^*, as they are known to suppress position effect variegation and hence can modulate epigenetic circuitry [24]. Comparison of the eye colour of the progeny from these crosses with the original 3L-L transgenic lines was done visually under the microscope as well as by eye pigmentation assay (figures 5). The results are also tabulated in Table 2.We observed an increase in the eye colour of progeny for both the 3L-L transgenic Drosophila lines when they were crossed to one or more of the Polycomb mutants. Both, 25.2.12 and25.2.29, transgenic lines showed eye colour increase in crosses with *Pc^1^*, *Asx^XF53^* and *Pho*. In addition, 25.2.29 also showed eye color increase when crossed with *Phd*, *esc^2^* and *Su(z)2*. Progeny of both the 3L-L transgenic Drosophila lines also showed increase in eye colour upon crossing with *Su(var)2-5^01^*. On the other hand, none of the Trithorax mutants showed any change in the eye colour when crossed to the 3L-L transgenic Drosophila lines (figure 5A and Table 2).

**Table 2:** Interaction of DNMT3L DMC with Polycomb and Trithorax proteins.

LINE			25.2.12	25.2.29
Mutants	Protein Group	Protein Subgroup		
***Pc^1^***	Polycomb	PRC1	✓	✓
***Psc^1^***	Polycomb	PRC1	×	×
***Scm^R5-13B^***	Polycomb	PRC1	×	×
***Phd***	Polycomb	PRC1	×	✓
***su(z)2^1.a1^***	Polycomb	PRC1	×	✓
***esc^2^***	Polycomb	PRC2	✓	✓
***Pcl^T1^***	Polycomb	PRC2	×	×
***Asx^XF53^***	Polycomb	Polycomb related	✓	✓
***Pho***	Polycomb	PhoRC	✓	✓
***Su(var)3-9^06^***	Suppressor of variegation		×	×
***Su(var)2-5^01^***	Suppressor of variegation		✓	✓
***Ash2^1^***	Trithorax	MLL complex	×	×
***Mor^1^***	Trithorax	Swi/snf complex	×	×
***Brm^2^***	Trithorax	Swi/snf complex	×	×
***Trl^R85^***	Trithorax	Trx recruiter	×	×
***Trx^1^***	Trithorax	TAC1 complex	×	×

Comparison of eye color of DNMT3L DMC transgenic lines with progeny of their crosses with Polycomb, Trithorax and Suvar mutants was performed. Progeny from the crosses showing eye color change are denoted by ✓. × represents no change.

To confirm that the effect on *mini-white* gene expression was due to the interaction of Polycomb proteins with DNMT3L DMC and did not reflect the chromatin organisation of the genomic loci where the transgene had got integrated, crosses of Polycomb mutants were set up with the 3L-L flipped out lines. As shown in Figure S2, no significant difference in eye colour was noted in the progeny of the crosses between Polycomb mutants and 3L-L flipped out lines indicating that the repression of mini-white gene was being mediated by the interaction of polycomb group of proteins with the 3L-L region. To further confirm the binding of Polycomb protein to the 3L-L region in the transgenic lines, we performed ChIP with Pc′ antibody and analysed for Pc′ binding with 3L-L region in the two independent 3L-L transgenic lines (25.2.12 and 25.2.29). As can be seen in Figure S3, Polycomb protein indeed binds to the 3L-L region.

### Histone modification profile of the Hsp70 promoter in the mini-white reporter construct in the presence of DNMT3L DMC

**Figure 6:**
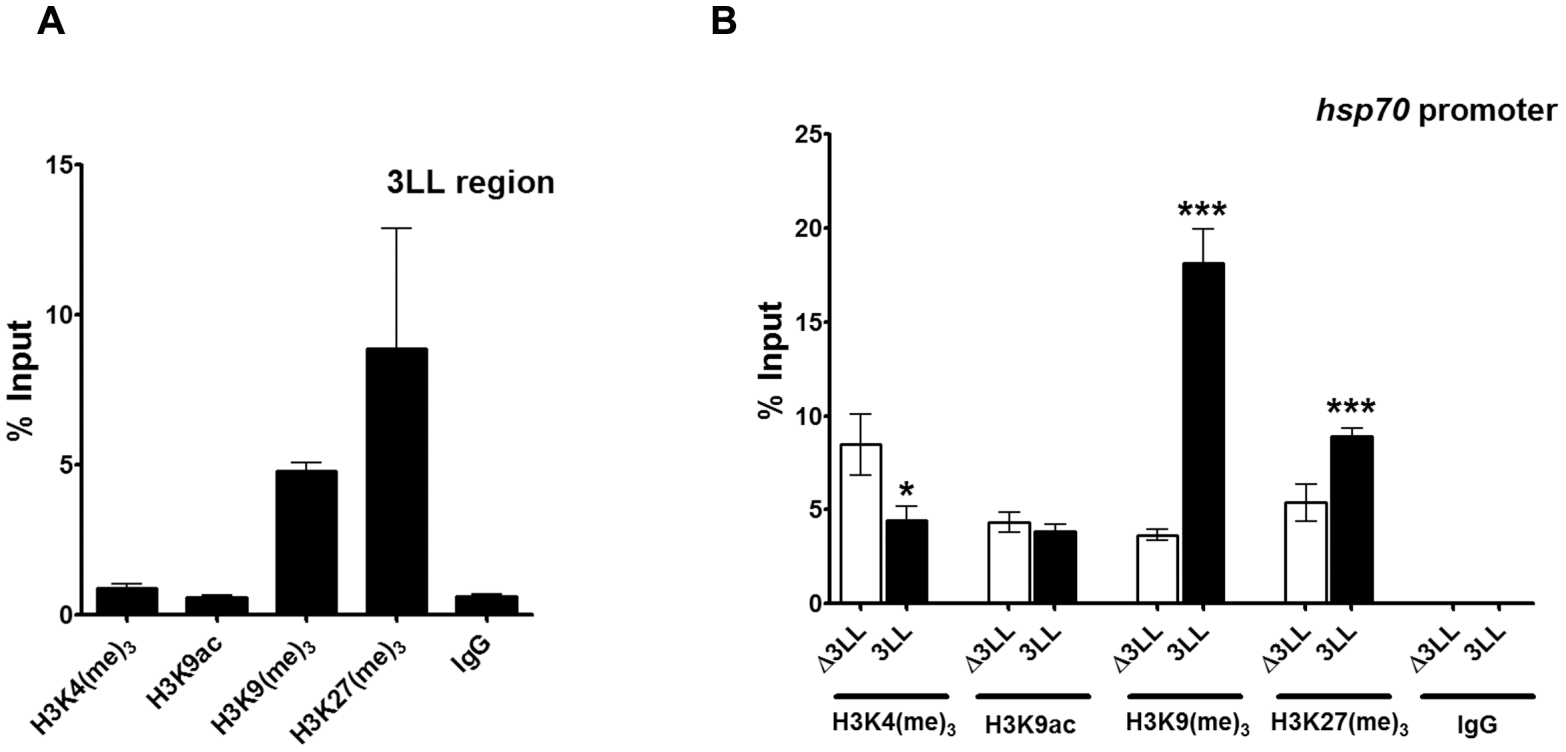
Epigenetic profile of DNMT3L DMC and the associated promoter in the reporter gene assay. A&B. Histone modifications associated with DNMT3L DMC and the hsp70 promoter in the transgene reporter assay in Drosophila. Histone ChIP analysis for the 3L-L region (A) and the CMV promoter (B) in the Drosophila transgene assay. Chromatin immunoprecipitation was carried out on the 25.2.12 transgenic line with the indicated histone H3 modifications, followed by quantitative Real-time PCR. Comparison of histone modifications associated with the hsp70 promoter in the 3L-L (3LL, black bars) transgenic lines and their flipped out counterparts (Δ3LL, white bars). The H3 histone modifications examined are mentioned below the X-axis. Enrichment in the bound fraction is represented as percentage of Input. IgG - control ChIP with rabbit IgG. Error bars represent Standard Deviation (S.D.). Asterisks indicate significant difference (Student's t test, * - p<0.05, ** - p<0.01, *** - p<0.005).

Several of the Polycomb group of proteins and Suvar proteins are involved in modulating chromatin conformation either by modifying or binding to specific modified residues on histone proteins [4], [29], [30]. The modifications of histone that are associated with a locus reflect its chromatin conformation [30]. To examine whether the interaction of Polycomb group of proteins with the DNMT3L DMC translated in to change in chromatin conformation at the *hsp70* promoter and 3L-L region of the reporter gene construct, we probed the association of some histone modifications to these regions. Using antibodies to H3K4me_3_ and H3K9ac (specific to active chromatin) and H3K9me_3_ and H3K27me_3_ (usually associated with inactive chromatin) we performed Chromatin Immunoprecipitation analyses on the 25.2.12 DNMT3L DMC transgenic line [31], [32]. As can be seen in figure 6A, the 3L-L region was preferentially associated with H3K9me_3_ and H3K27me_3_ as compared to the H3K4me_3_ and H3K9ac modifications (p<0.05). Comparison of these modifications between the *hsp70* promoter in the 3L-L transgenic lines and their flipped out counterpart showed that H3K9me_3_ and H3K27me_3_ association with the *hsp70* promoter was significantly more in the 3L-L lines than their flipped-out counterpart (p<0.005 for H3K9me_3_ and p<0.01 for H3K27me_3_, figure 6B). H3K4me_3_, an active chromatin specific histone modifications was significantly less associated with the *hsp70* promoter in the 3L-L line as compared to its flipped-out counterpart (p<0.05, figure 6B). No change was observed for the association of H3K9ac with *hsp70* promoter in the two lines.

### Effect of DNMT3L DMC on the interaction between the CMV promoter and Polycomb/Trithorax proteins in mammalian cells

**Figure 7:**
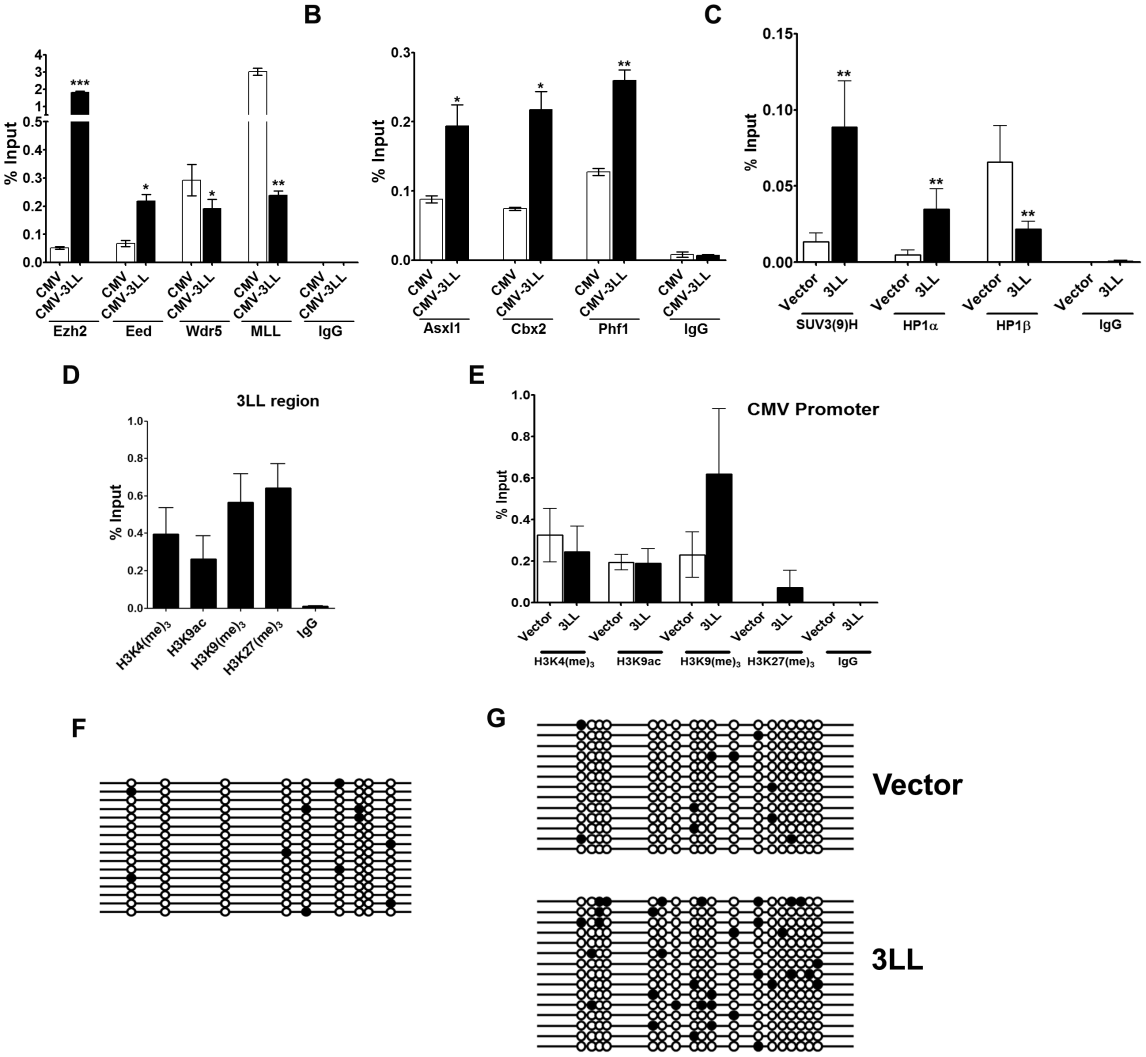
Epigenetic profile of the 3L-L region and CMV promoter in transient transfection assay. A–C. Effect of DNMT3L DMC on the interaction between CMV promoter and Polycomb, Trithorax and Suvar proteins in mammalian cells. ChIP analysis for the CMV promoter in control (referred to as CMV) and 3L-L region (CMV-3LL) containing mammalian transient transfection constructs (see figure 2A for graphical representation of the constructs). Quantitation was done by Real-time PCR. Enrichment of the CMV region in the bound fraction is represented as percentage of Input. The names of the chromatin proteins examined are mentioned below the X-axis. D–G. Histone modifications and DNA methylation profile of the 3L-L region in the *AcGFP* reporter gene construct after transient transfection in HEK293 cells. C- ChIP analysis for the 3L-L region with the indicated histone H3 modifications. D- Comparison of histone modifications associated with the CMV promoter in CMV only (vector) and 3L-L (3LL) constructs after transfection into HEK293 cells. Enrichment in the bound fraction is represented as percentage of Input. IgG - control ChIP with rabbit IgG. Error bars represent Standard Deviation (S.D.). Asterisks indicate significant difference (Student's t test, * - p<0.05, ** - p<0.01, *** - p<0.005). F, G- Methylation profile of the 3L-L region (F) and CMV promoter (G) in the construct after bisulfite sequencing. Compare the methylation profile of the CMV promoter in CMV only (vector) and the 3L-L (3LL) constructs. Each horizontal line indicates a single clone from the 3L-L PCR products after bisulfite treatment. Circles denote CpG dinucleotides present within the sequence. The positions are not drawn to scale. Open circles indicate no methylation. Filled circles represent methylated cytosine.

Based on our observation that the 3L-L region interacted with the Polycomb proteins in the Drosophila reporter gene assay, we sought to examine the interaction of various Polycomb and Trithorax proteins with the CMV promoter in the mammalian transient transfection reporter assay by ChIP analysis using antibodies to these proteins. Polycomb proteins EZH2, EED, ASXL1, CBX2 and PHF1; Trithorax proteins MLL and WDR5; Histone methyltrasferase SUV39H1 and the heterochromatin proteins HP1α (CBX5, product of *Su(var)2-5* in Drosophila) and HP1β (CBX1) were analysed for their association with the CMV promoter [24]. Similar to our observations in the Drosophila experiments, the association of Polycomb proteins EZH2, EED, ASXL1, CBX2 and PHF1 was significantly more with the CMV promoter in presence of the 3L-L region (figure 7 A & B). The association of both the Trithorax proteins WDR5 and MLL were significantly less with the CMV promoter in the presence of 3L-L region in the construct (figure 7A). SUV39H1 and HP1α also associated significantly more with the CMV promoter in the presence of the 3L-L region (Figure 7C). However, HP1β showed decreased association with CMV promoter in presence of the 3L-L region in the construct (Figure 7C).

### Histone modification and DNA methylation profile of the CMV and 3L-L region in the transient transfection assay

To examine if the epigenetic status of the 3L-L region and the CMV promoter is also influenced by the presence of the 3L-L region, we performed Chromatin immunoprecipitation (ChIP) analysis on transiently transfected HEK293 cells using antibodies against various Histone modifications. As shown in figure 7D, the 3L-L region in the 3L-L+ construct showed a preferential association with H3K9me_3_ and H3K27me_3_ modifications as compared to H3K9ac. Comparison of ChIP analysis for the CMV promoter in the 3L-L+ and the CMV only constructs showed a gain of H3K9me_3_ and H3k27me_3_ modifications at the CMV promoter in the 3L-L+ constructs (figure 7E). No difference was observed between the 3L-L+ and control constructs for the H3K4me_3_ and H3K9ac modifications (figure 7E).

Unlike Drosophila, DNA methylation is an important part of the epigenetic circuitry in mammalian cells [33]. To assess if the presence of 3L-L in the construct has any effect on the DNA methylation status of the CMV promoter we performed DNA methylation analysis by Bisulfite sequencing. No significant difference was observed in the DNA methylation profile of the CMV promoter between the cells transfected with the control CMV and the 3L-L+ construct (figure 7G). The 3L-L region (figure 7F) in the 3L-L+ construct also showed no appreciable gain of DNA methylation in the transient transfection assay.

### Epigenetic profile of the DNMT3L DMC at the endogenous locus in mammalian cells

**Figure 8:**
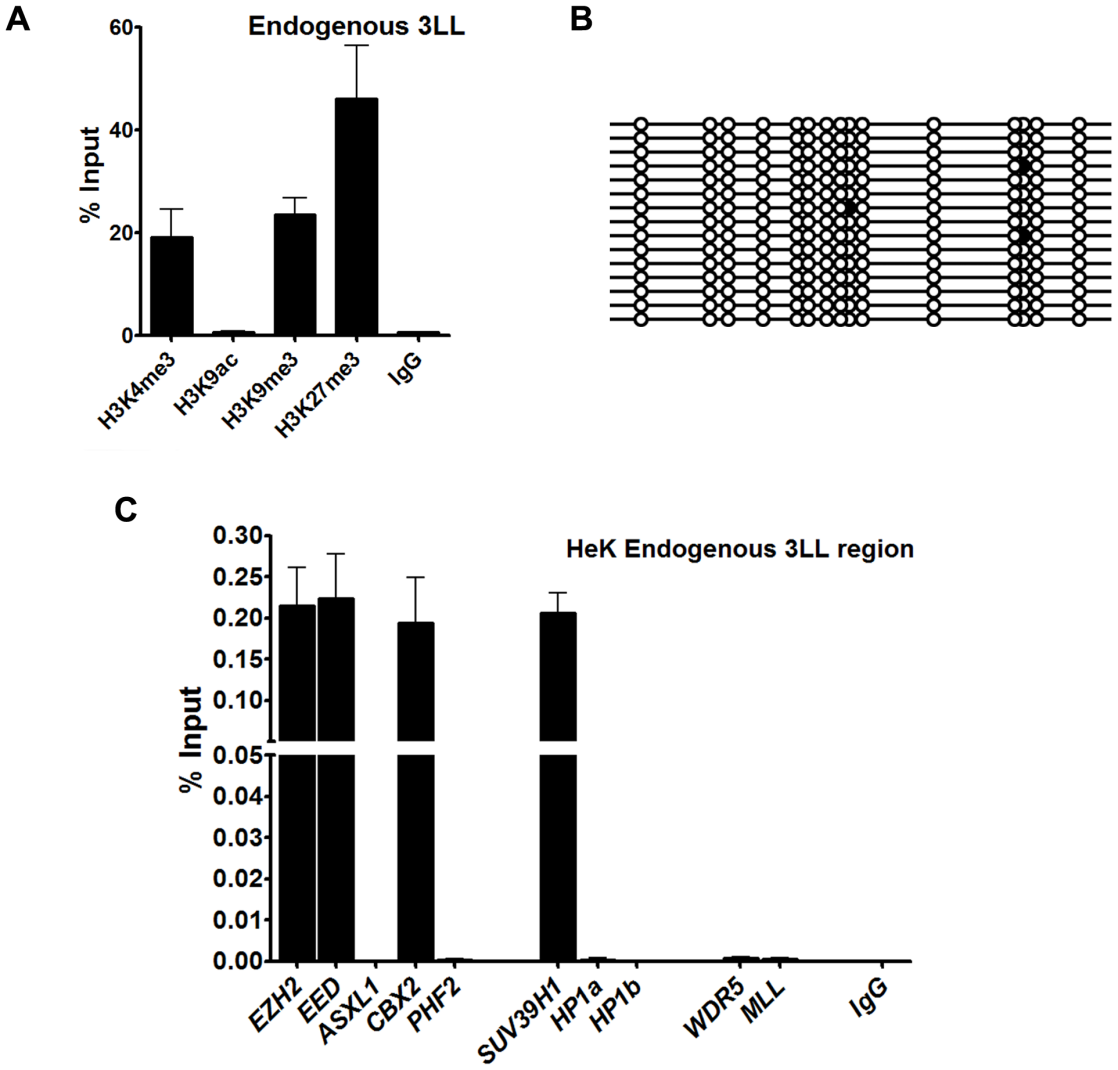
Epigenetic profile of endogenous DNMT3L DMC. A- Histone modifications; B. DNA methylation profile of the 3L-L region at the endogenous *DNMT3L* locus in HEK293 cells. C. ChIP analysis for the 3L-L region at the endogenous *DNMT3L* locus using antibodies to the various Polycomb, Trithorax and Suvar proteins mentioned below the X-Axis. Enrichment in the bound fraction is represented as percentage of Input. IgG - control ChIP with rabbit IgG. Error bars represent Standard Deviation (S.D.). Asterisks indicate significant difference (Student's t test, * - p<0.05, *** - p<0.001, *** - p<0.005).

Next it was sought to assess whether the epigenetic status of the DNMT3L DMC region in its endogenous locus matched with what was observed in the reporter gene assays. As was observed for the 3L-L region in the transfection construct, endogenous 3L-L region in the HEK293 cell line (used for the transient transfection assays) also preferentially associated with H3K9me_3_ and H3K27me_3_ as compared to H3K9ac (figure 8A). In addition, DNA methylation status of the DNMT3L DMC region at the endogenous locus was found to be unmethylated in HeK cells, similar to what was observed for the 3L-L region in the construct (figure 8B).

To test whether the histone modifications observed at the endogenous 3L-L region correlated with its interaction to relevant Polycomb and Suvar group proteins, we performed ChIP with antibodies against a few Polycomb, Suvar and Trithorax group of proteins for the DNMT3L DMC region at the endogenous locus in HeK cells. As can be seen from figure 8C for HeK cells, DNMT3L DMC preferentially associated with EZH2, EED, CBX2 (Polycomb) and SUV39H1 (Suvar) proteins. Both the Trithorax proteins MLL and WDR5 did not show any association with DNMT3L DMC. This interaction profile of DNMT3L DMC with Polycomb and Suvar proteins was not dependent of its DNA methylation status as we observed the same profile for HeLa cells that showed higher level of DNA methylation (Figure S4A, B). However, as reported previously [15], *DNMT3L* expression seems to be partially dependent on DNA methylation as its expression was found to be more in HeK293 cells as compared to HeLa cells (Figure S4C).

### Effect of Polycomb proteins on the transcription of the *DNMT3L* gene from the endogenous locus

**Figure 9:**
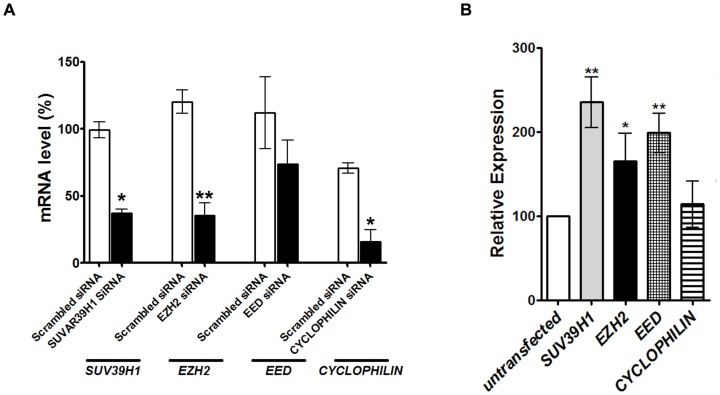
Knock-down of Polycomb proteins and its effect on transcription of the *DNMT3L* gene from the endogenous locus. A. siRNA mediated transcriptional repression of polycomb proteins. mRNA levels of the indicated Polycomb genes was assayed in presence of scrambled or specific siRNA in HEK293 cells. % mRNA level was calculated with respect to untransfected cells. B. *DNMT3L* gene expression from the endogenous locus in Polycomb siRNA transfected or untransfected HEK293 cells was quantitated by Real-time RT-PCR. Relative expression was calculated with respect to untransfected cells. The genes for which siRNA was used in our assay are mentioned below the X-axis. Error bars represent Standard Deviation (S.D.). Asterisks indicate significant difference (Student's t test, * - p<0.05, *** - p<0.001, *** - p<0.005).

To examine whether Polycomb proteins have an effect on the transcription of the *DNMT3L* gene from its endogenous locus in HEK293 cells, siRNA based knock-down of *SUV39H1*, *EZH2* and *EED* was performed. Approximately 70% decrease in expression of *SUV39H1*, *EZH2* and control *CYCLOPHILIN B* gene and approximately 35% decrease in *EED* expression was observed (figure 9A). Comparison of *DNMT3L* expression between untransfected HEK293 cells and HEK293 cells transfected with the various siRNAs was performed by Real-time RT-PCR. As can be seen in figure 9B, statistically significant increase in *DNMT3L* expression was observed in HEK293 cells transfected with siRNA against *SUV39H1*, *EED* (p<0.01) and *EZH2* (p<0.05) as compared to untransfected cells. *CYCLOPHILIN* B siRNA had no effect on *DNMT3L* expression.

## Discussion

The role of *DNMT3L* in modulating the DNA methylation at several imprinted loci and its interactions with various epigenetic modifiers like the de novo DNA methyltransferases DNMT3A & DNMT3B and histone H3 at Lysine 4, confers it an important role in regulation of mammalian development [9], [13]. Previous results from our laboratory indicated that overexpression of the human *DNMT3L* gene was correlated with carcinogenesis [15], [16]. This would indicate that *DNMT3L* transcription needs to very tightly regulated so that it is kept silent in most somatic cell types and expressed at appropriate levels only in germ cells and during early embryogenesis [13], [14]. In our present study we show a Polycomb/Trithorax Response Element (PRE), present within the CpG island that encompasses the *DNMT3L* promoter and first exon region (DNMT3L DMC), can modulate its expression.

### DNMT3L DMC is a repressor of transcription

DNMT3L DMC is the same region which was shown to be hypomethylated in cervical and ocular cancer samples [16], [17]. The loss of DNA methylation observed for this region was found to be correlated with increased expression of *DNMT3L*, which in turn was found to be a cause of nuclear reprogramming, a salient characteristic of carcinogenesis [15]. There is a possibility that the loss of methylation at DNMT3L DMC observed in cancer samples was merely a reflection of nuclear reprogramming observed in cancer [34]. But it is also possible that the changed DNA methylation profile of the *DNMT3L* locus indicated its role in carcinogenesis. If latter was the case then the reason for the loss of DNA methylation only within the promoter/first exon of *DNMT3L* could indicate an important role for this region in the regulation of *DNMT3L*. We show through both Drosophila and mammalian reporter gene assays, that the presence of DNMT3L DMC in cis acts to repress transcription of the reporter gene. The extent of the repression of GFP expression due to the presence of DNMT3L DMC in the transient transfection of mammalian cell was similar to that observed for H19 ICR, a known transcriptional repressor [19]. The ability of DNMT3L DMC to repress transcription and the fact that *DNMT3L* is kept transcriptionally silent in most somatic cell types would suggest a role for DNMT3L DMC in the tight regulation of *DNMT3L* transcription [13], [14].

Dissection of the DNMT3L DMC into smaller regions showed that a 80 bp region was sufficient to repress reporter gene expression (figure 3). We could identify one binding site for the polycomb protein Pho and 5 binding sites for YY1, a mammalian homolog of Pho within the DNMT3L DMC region but none in the minimal 80 bp region [35]. Interestingly, all the overlapping region of 3L-S that were used in our reporter gene assay showed varying levels of repression. Transcriptional repression by s1+ was not to the same extent as 3L-S but it still was able to weakly repress the reporter gene transcription (approximately 20% repression). This would indicate presence of multiple cis-elements or motifs within DNMT3L DMC that are capable of transcriptional repression.

### DNMT3L DMC is a Polycomb Response Element (PRE)

In Drosophila, the role of Polycomb and Trithorax group of proteins has been well characterised in the developmental control of Hox gene expression [32]. Polycomb proteins are involved in inhibiting Hox gene expression while Trithorax proteins are involved in maintaining Hox genes in an active state [26]. Both the transgenic DNMT3L DMC Drosophila lines, the 3L-L region in the reporter gene construct and the endogenous 3L-L showed interaction with several members of Polycomb proteins that we tested. This suggested that the inhibition of the *mini-white* reporter gene in the DNMT3L DMC transgenic flies was being mediated through Polycomb proteins. The role of Polycomb proteins in repression of *DNMT3L* gene expression was further confirmed by knock-down of Polycomb proteins in mammalian cells which resulted in increased expression of endogenous *DNMT3L*.

Polycomb proteins can be sub-classified into at least three groups, PRC1, PRC2 and PhoRC, based on which repressive complex they are part of. While *Pho* is the DNA binding component of the Polycomb complexes, PRC1 and PRC2 complexes achieve chromatin compaction and repression by different mechanisms [36],[37]. DNMT3L DMC in Drosophila transgenic lines and transient transfection assay was able to interact with members of all the three Polycomb subgroups (Table 2, Figure 7). Apart from Polycomb and Trithorax, proteins like HP1α (su(var)2-5) and SUV39H1 are also involved in chromatin organisation. SUV39H1 is a H3K9 methytransferase and Heterochromatin associated HP1α is generally associated with gene repression and bind to H3 at lysine K9 in the trimethylated form [24], [38], [39]. HP1α also form a complex with SUV39H1 [40]. Our results show that HP1α and SUV39H1 also interact with DNMT3L DMC leading to inhibition of the reporter gene expression.

It has been suggested that regions that show interaction with both PRC1 and PRC2 complexes have epigenetic regulatory properties and are part of large CpG islands [41]–[43]. DNMT3L DMC, which is part of a CpG island, is methylated in most somatic tissues [15]–[17] and we show here it interacts with PRC1, PRC2 and PhoRC polycomb repressive complexes. This would indicate that DNMT3L DMC possess essential attributes of a regulatory element. That this CpG island within the *DNMT3L* gene possesses multiple cis-elements/motifs, is normally methylated and interacts with multiple repressive elements also underlines its importance in keeping a tight control on the transcription level of *DNMT3L* in various cell types.

It was interesting to note decreased association of HP1β (CBX1) with the CMV promoter in presence of DNMT3L DMC. While both the HP1 (HP1α and HP1β) homologs have been correlated with repression of gene expression especially in heterochromatin context, there have been a few reports that have indicated a role of HP1β (or Drosophila HP1b) in transcriptional activation [44]–[46].

### Presence of DNMT3L DMC causes the adjacent promoter to adopt an inactive chromatin conformation

Regulation of gene activity is achieved by the interplay of DNA with histones and non-histone proteins within the chromatin context [30]. Polycomb group of proteins are either enzymes that can modify specific residues of the histone tails or are proteins that bind to these modified residues [4]. Concordant with the observation of its interaction with Polycomb proteins, our results also showed that DNMT3L DMC adopts an inactive chromatin conformation in both the Drosophila transgene reporter gene assay and the mammalian transient transfection assay. In both assays, DNMT3L DMC region associated preferentially with inactive chromatin associated histone modifications H3K9(me)_3_ and H3K27(me)_3_ as compared to H3K9ac, a modification linked to active chromatin [31]. Importantly, the presence of the DNMT3L DMC in the construct influenced the chromatin organisation of the reporter gene promoter. In both Drosophila and mammalian cells, the promoter for the reporter gene showed a gain of inactive chromatin-specific histone marks. This indicated that the inactive chromatin conformation adopted by DNMT3L DMC had either spread to the promoter or had influenced the promoter into adopting an inactive chromatin conformation. The inactive chromatin profile at the DNMT3L DMC region within the reporter gene constructs was also mirrored at the endogenous locus in HEK293 cells.

Finally, the importance of identifying a cis-regulatory element (a PRE) with in the DNMT3L gene should be viewed in light of the loss of DNA methylation at the DNMT3L DMC observed in certain cancers [16], [17]. *DNMT3L* is an epigenetic effector that provides specificity to the DNA methylation activity of de novo methyltransferases, plays an important part in setting up DNA methylation imprints in the germ cells and is associated with nuclear reprogramming during carcinogenesis [11], [15]. Because of its important role in regulating the epigenetic circuitry, our findings that multiple layers of epigenetic modifications at DNMT3L DMC are being utlilised to repress *DNMT3L* expression support our hypothesis that tight regulation of *DNMT3L* expression is required to avoid nuclear reprogramming and initiation of carcinogenesis. Therefore, epigenetic changes at the *DNMT3L* locus leading to its overexpression could be an important event in carcinogenesis. However, further work would be required to identify the regulatory framework for *DNMT3L* in cancer patients and establish the role of this regulatory element during carcinogenesis.

## Supporting Information

Figure S1 **ChIP analysis for known region in the human genome with the antibodies against histone modifications that we used in our study.** The efficacy of the antibodies in our Chromatin immuneprecipitation assay was checked by performing ChIP for genomic loci that are known to be associated with the tested histone modifications. Enrichment in the bound fraction is represented as percentage of Input. The loci tested and the histone modifications examined are mentioned below the X-axis. IgG - control ChIP with rabbit IgG. Error bars represent Standard Deviation (S.D).
(TIF)

Figure S2 **DNMT3L DMC interacts with Polycomb group of proteins.** Comparison of eye color pigmentation between Δ3L-L transgenic lines (25.2.12 and 25.2.29) and their counterpart lines after crossing with the respective Polyomb (*Pc^1^*, *esc^2^*, *Asx^XF53^*, *Psc′*, *Pcl^T1^*, *Scm^R5-13B^*, *Phd*, *Pho*), Trithorax (*Ash2^1^*, *Mor^1^*, *Bbrm^2^*, *Trl^R85^*, *Trx^1^*) and Supressor of Variegation (*Su(var)2-5^01^*, *Su(var)3-9^06^*) mutants. Δ P/+, heterozygous Δ 3L-L transgenic lines; Δ 3LL/−, their respective counterparts after crosses with the respective mutant (− is the name of the Polycomb, Trithorax or Suvar mutant). Each bar represents eye color pigmentation for progeny from crosses of individual transgenic lines with a particular mutant, the details of which are provided below the X-axis. As the assays were done in batches, the eye pigmentation for the control 3L-L transgenic line was done for each batch and is shown as white bars (ΔP/+ male with *W^1118^*) in the graphs. Error bars represent Standard Deviation (S.D.). Asterisks indicate significant difference (Student's t test, * - p<0.05, ** - p<0.01).
(TIF)

Figure S3 **ChIP analysis for the 3L-L region in the reporter gene construct from transgenic Drosophila.** ChIP analysis was done using Polycomb protein antibodies for the 3L-L region in the reporter gene construct. Quantitation was done by Real-time PCR. Enrichment of the 3L-L region in the bound fraction is represented as percentage of Input. IgG - control ChIP with rabbit IgG. Error bars represent Standard Deviation (S.D.).
(TIF)

Figure S4 **Epigenetic profile of endogenous DNMT3L DMC in HeLa cells.** A- ChIP analysis for the 3L-L region at the endogenous *DNMT3L* locus using antibodies to the various Polycomb, Trithorax and Suvar proteins mentioned below the X-Axis. Enrichment in the bound fraction is represented as percentage of Input. IgG - control ChIP with rabbit IgG. B. DNA methylation profile of the 3L-L region at the endogenous *DNMT3L* locus in HeLa cells. C. *DNMT3L* gene expression from the endogenous locus in HeLa and HEK293 cells was quantitated by Real-time RT-PCR. Error bars represent Standard Deviation (S.D.).
(TIF)

Table S1 **Genomic location of the 3L-L construct in transgenic Drosophila.** Inverse PCR followed by Sequencing of the PCR product was done to determine the Drosophila genomic loci harbouring the 3L-L construct. The coordinates mentioned are with reference to the Drosophila genomic sequence available in NCBI (provided in column 5).
(XLSX)

Supplementary Information S1 **Isolation of chromatin from Drosophila larvae.** Section 1 describes protocol to isolate Drosophila Larvae chromatin. Section 2 describes protocol to perform Chromatin Immunoprecipitation on Drosophila Larvae.
(DOC)
