# Correction: The CpG Island Encompassing the Promoter and First Exon of Human *DNMT3L*Gene Is a *PcG/TrX* Response Element (PRE)

**DOI:** 10.1371/journal.pone.0105714

**Published:** 2014-08-12

**Authors:** The PLOS ONE Staff

The y-axis scales in Figure 6 and Figure 8A are incorrect. The authors have provided corrected versions of Figures 6 and 8 below.

**Figure 6:**
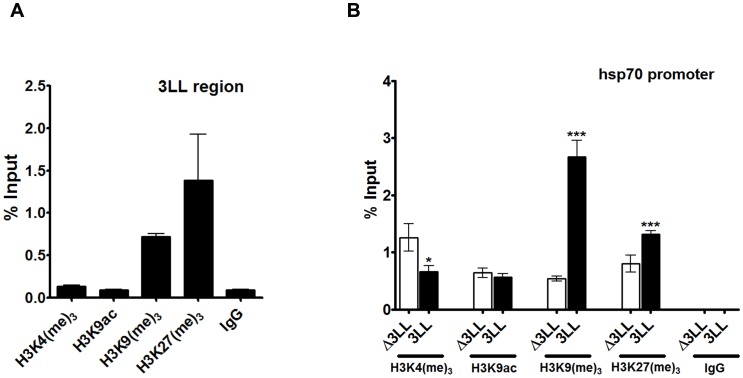
Epigenetic profile of DNMT3L DMC and the associated promoter in the reporter gene assay. A&B. Histone modifications associated with DNMT3L DMC and the hsp70 promoter in the transgene reporter assay in Drosophila. Histone ChIP analysis for the 3L-L region (A) and the CMV promoter (B) in the Drosophila transgene assay. Chromatin immunoprecipitation was carried out on the 25.2.12 transgenic line with the indicated histone H3 modifications, followed by quantitative Real-time PCR. Comparison of histone modifications associated with the hsp70 promoter in the 3L-L (3LL, black bars) transgenic lines and their flipped out counterparts (Δ3LL, white bars). The H3 histone modifications examined are mentioned below the X-axis. Enrichment in the bound fraction is represented as percentage of Input. IgG - control ChIP with rabbit IgG. Error bars represent Standard Deviation (S.D.). Asterisks indicate significant difference (Student's t test, * - p<0.05, ** - p<0.01, *** - p<0.005).

**Figure 8:**
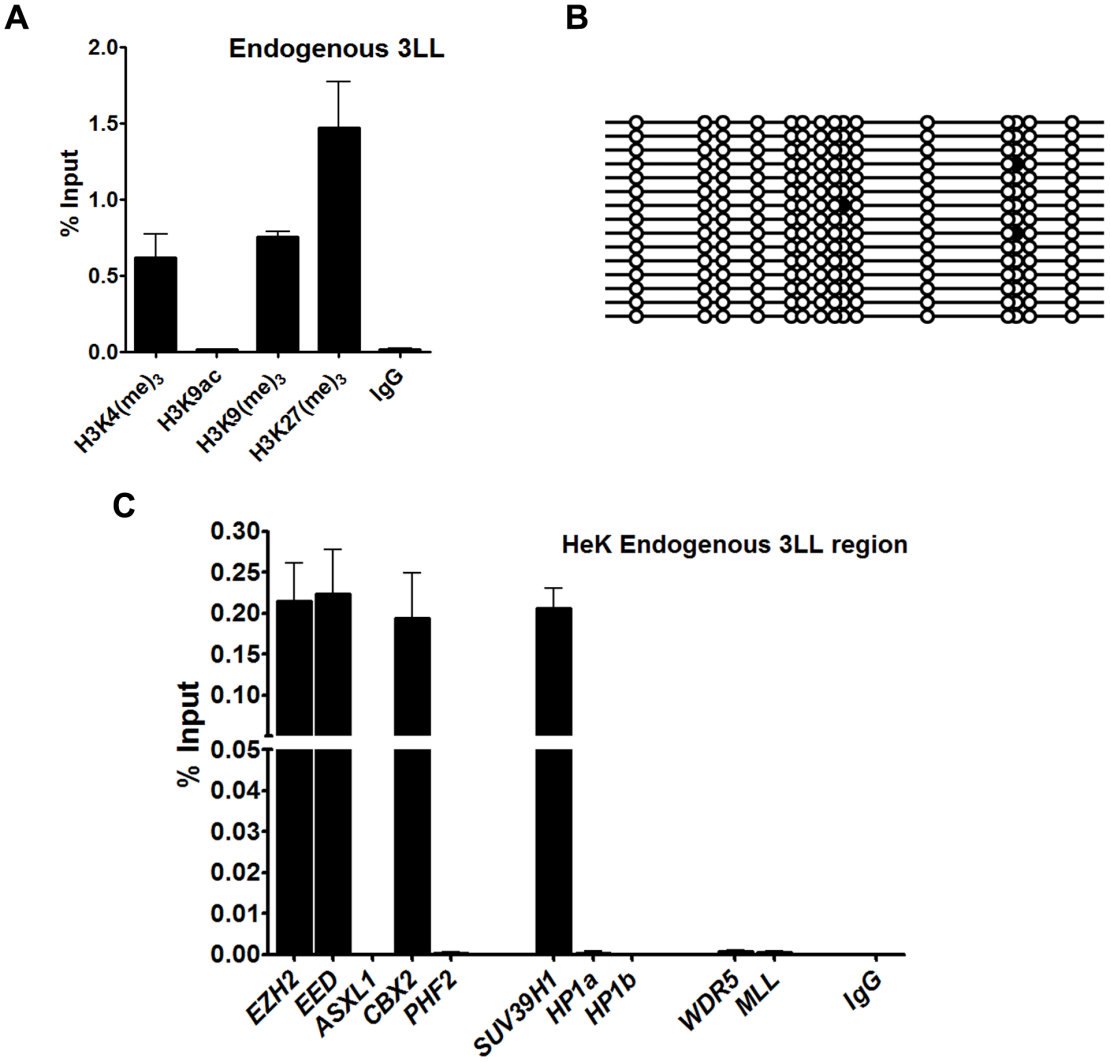
Epigenetic profile of endogenous DNMT3L DMC. A- Histone modifications; B. DNA methylation profile of the 3L-L region at the endogenous *DNMT3L* locus in HEK293 cells. C. ChIP analysis for the 3L-L region at the endogenous *DNMT3L* locus using antibodies to the various Polycomb, Trithorax and Suvar proteins mentioned below the X-Axis. Enrichment in the bound fraction is represented as percentage of Input. IgG - control ChIP with rabbit IgG. Error bars represent Standard Deviation (S.D.). Asterisks indicate significant difference (Student's t test, * - p<0.05, *** - p<0.001, *** - p<0.005).
