# Supplementary figures and images for: The CpG Island Encompassing the Promoter and First Exon of Human DNMT3L Gene Is a PcG/TrX Response Element (PRE)
Source: PLoS One. 2014 Apr 17;9(4):e93561. doi: 10.1371/journal.pone.0093561 (PMC3990577; doi:10.1371/journal.pone.0093561)

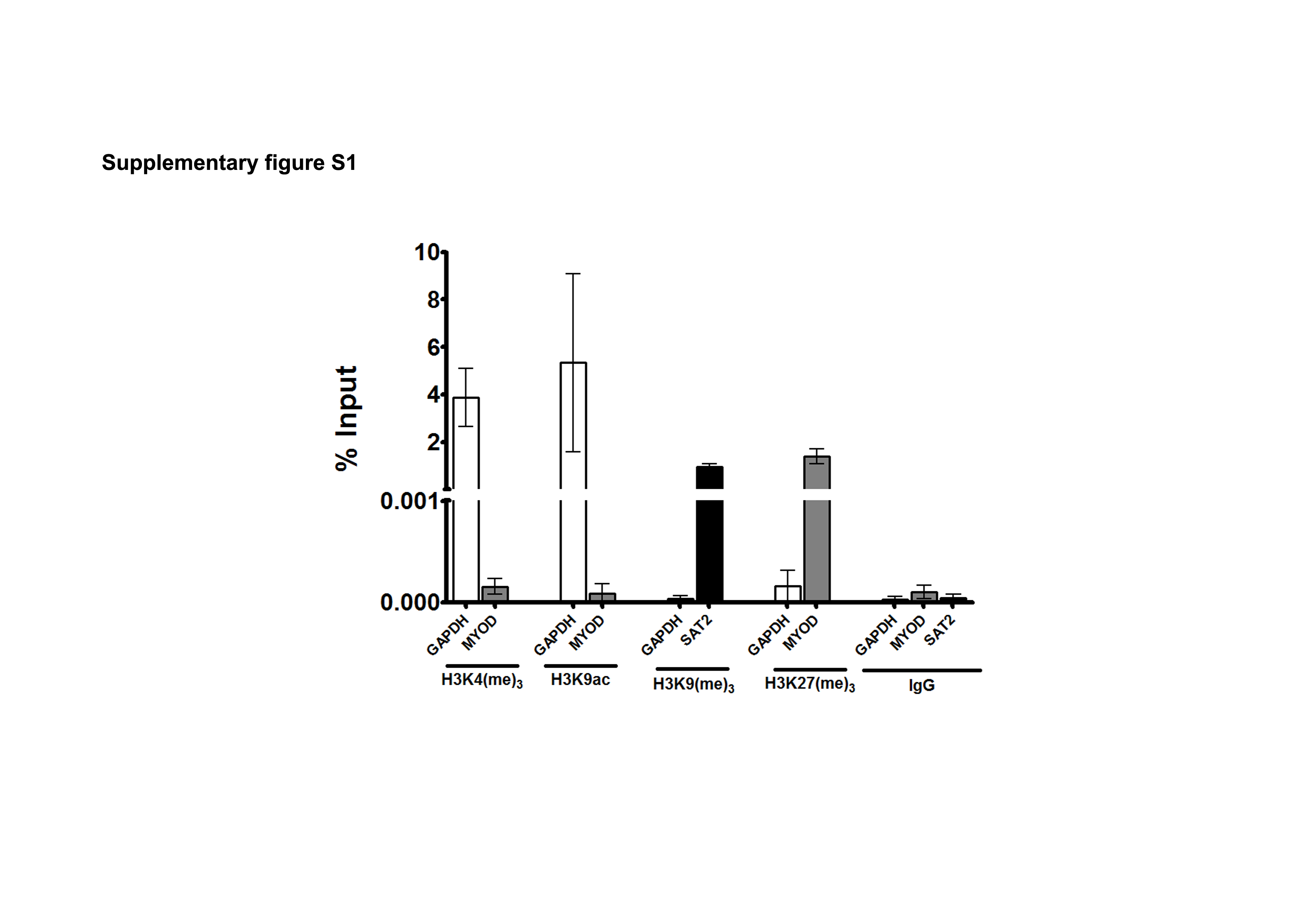

Supplement: Figure S1 — ChIP analysis for known region in the human genome with the antibodies against histone modifications that we used in our study. The efficacy of the antibodies in our Chromatin immuneprecipitation assay was checked by performing ChIP for genomic loci that are known to be associated with the tested histone modifications. Enrichment in the bound fraction is represented as percentage of Input. The loci tested and the histone modifications examined are mentioned below the X-axis. IgG - control ChIP with rabbit IgG. Error bars represent Standard Deviation (S.D). (TIF) [file pone.0093561.s001.tif]

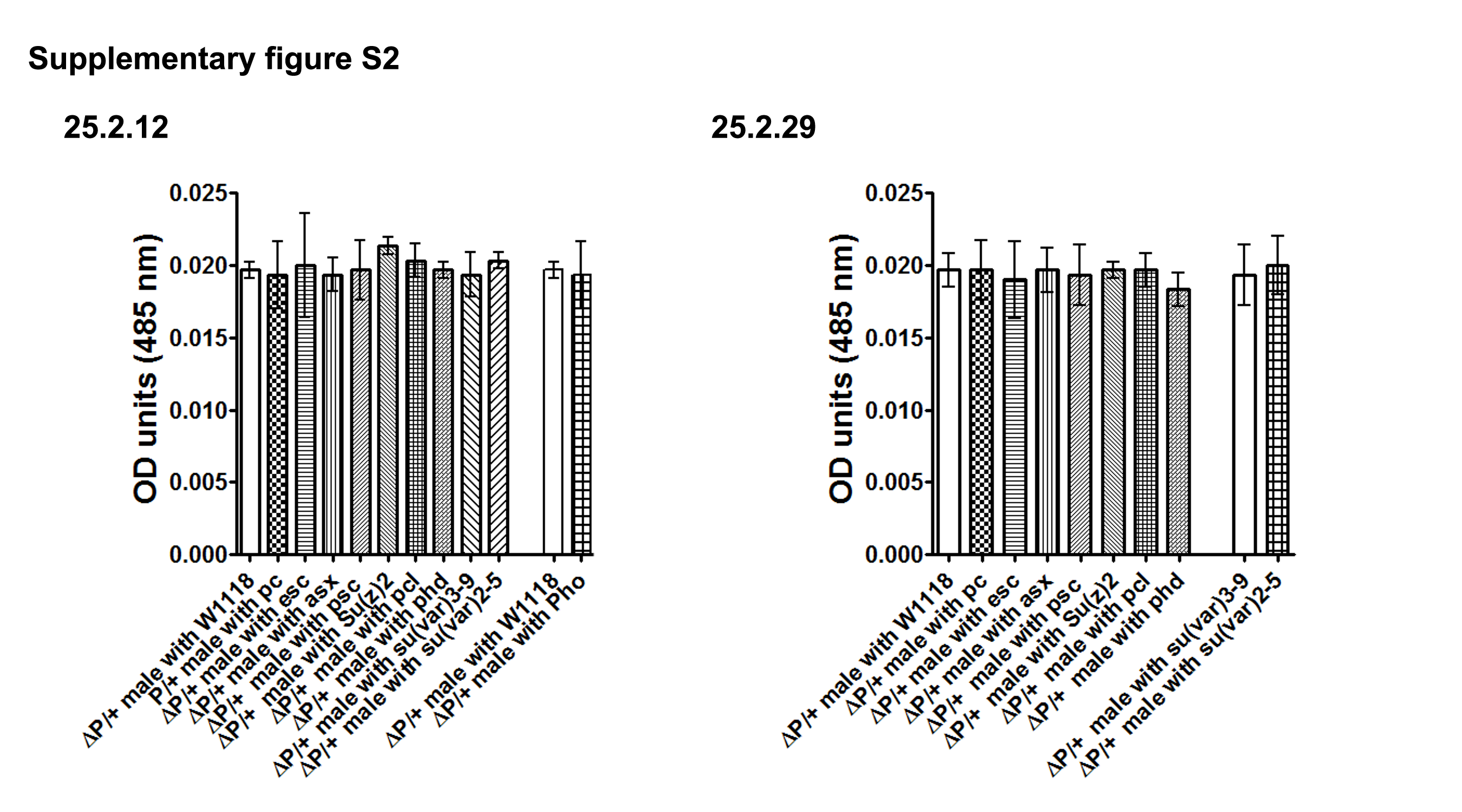

Supplement: Figure S2 — DNMT3L DMC interacts with Polycomb group of proteins. Comparison of eye color pigmentation between Δ3L-L transgenic lines (25.2.12 and 25.2.29) and their counterpart lines after crossing with the respective Polyomb (Pc1, esc2, AsxXF53, Psc′, PclT1, ScmR5-13B, Phd, Pho), Trithorax (Ash21, Mor1, Bbrm2, TrlR85, Trx1) and Supressor of Variegation (Su(var)2-501, Su(var)3-906) mutants. Δ P/+, heterozygous Δ 3L-L transgenic lines; Δ 3LL/−, their respective counterparts after crosses with the respective mutant (− is the name of the Polycomb, Trithorax or Suvar mutant). Each bar represents eye color pigmentation for progeny from crosses of individual transgenic lines with a particular mutant, the details of which are provided below the X-axis. As the assays were done in batches, the eye pigmentation for the control 3L-L transgenic line was done for each batch and is shown as white bars (ΔP/+ male with W1118) in the graphs. Error bars represent Standard Deviation (S.D.). Asterisks indicate significant difference (Student's t test, * - p<0.05, ** - p<0.01). (TIF) [file pone.0093561.s002.tif]

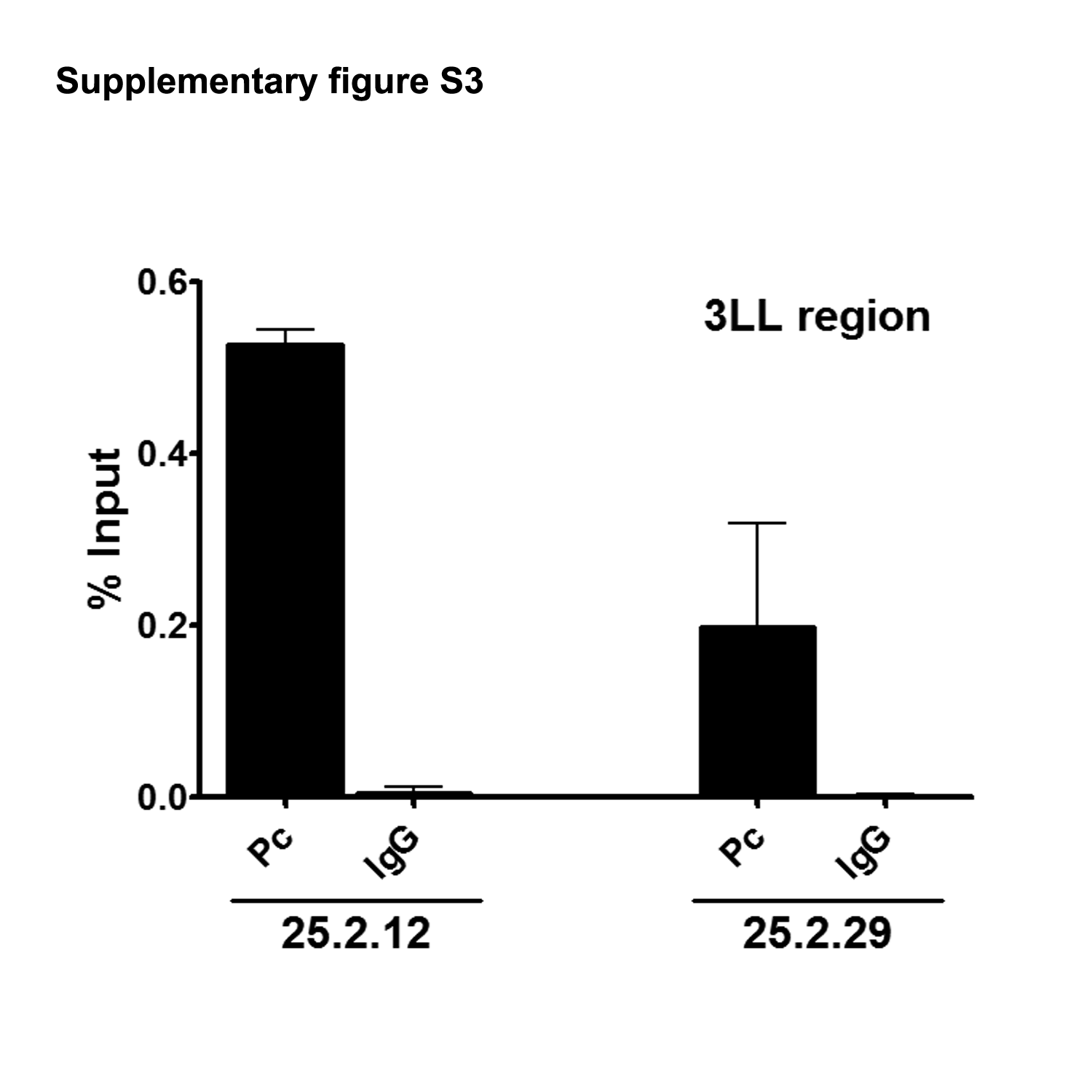

Supplement: Figure S3 — ChIP analysis for the 3L-L region in the reporter gene construct from transgenic Drosophila. ChIP analysis was done using Polycomb protein antibodies for the 3L-L region in the reporter gene construct. Quantitation was done by Real-time PCR. Enrichment of the 3L-L region in the bound fraction is represented as percentage of Input. IgG - control ChIP with rabbit IgG. Error bars represent Standard Deviation (S.D.). (TIF) [file pone.0093561.s003.tif]

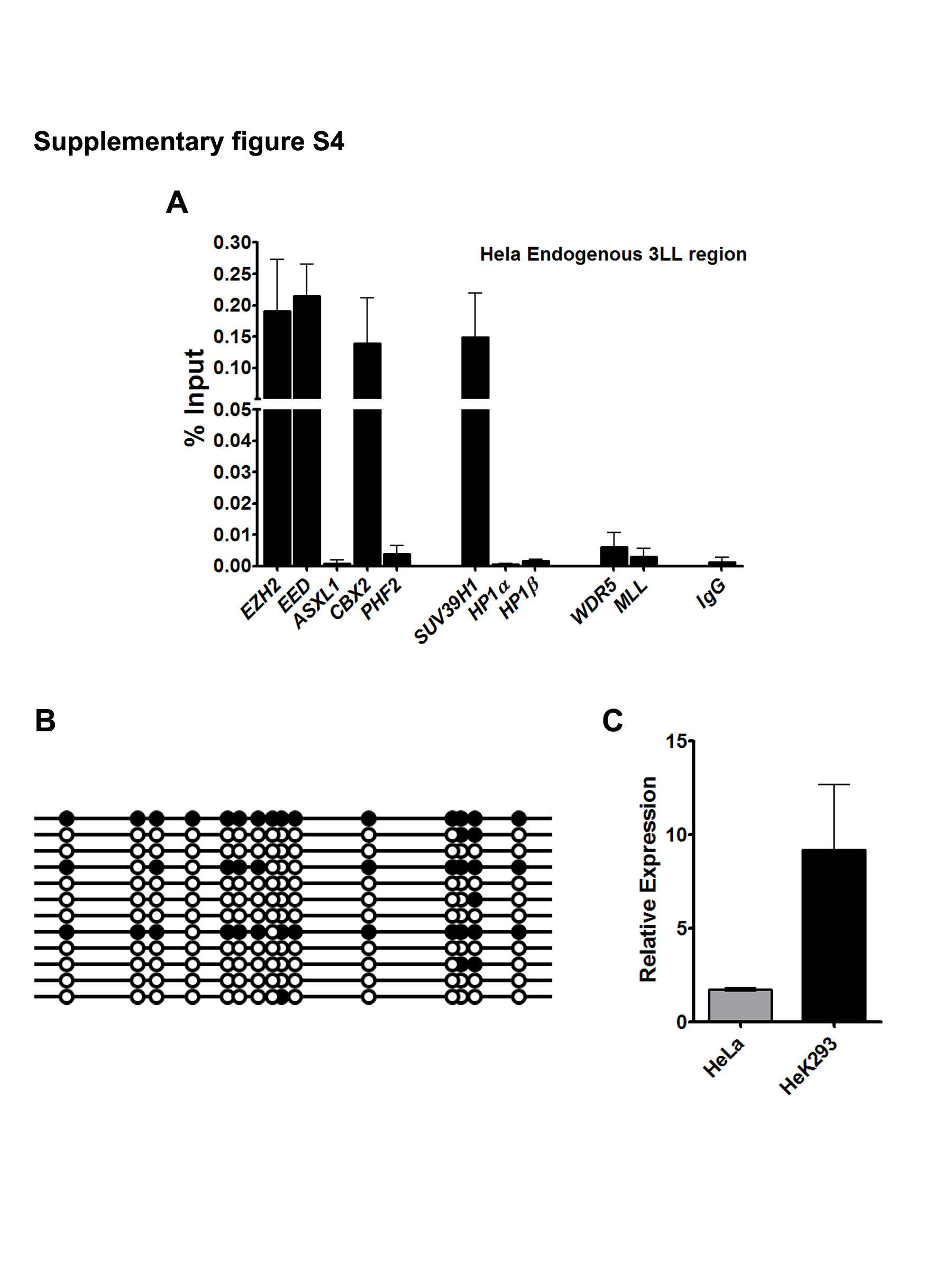

Supplement: Figure S4 — Epigenetic profile of endogenous DNMT3L DMC in HeLa cells. A- ChIP analysis for the 3L-L region at the endogenous DNMT3L locus using antibodies to the various Polycomb, Trithorax and Suvar proteins mentioned below the X-Axis. Enrichment in the bound fraction is represented as percentage of Input. IgG - control ChIP with rabbit IgG. B. DNA methylation profile of the 3L-L region at the endogenous DNMT3L locus in HeLa cells. C. DNMT3L gene expression from the endogenous locus in HeLa and HEK293 cells was quantitated by Real-time RT-PCR. Error bars represent Standard Deviation (S.D.). (TIF) [file pone.0093561.s004.tif]
