# Supplementary material for: The CpG Island Encompassing the Promoter and First Exon of Human DNMT3L Gene Is a PcG/TrX Response Element (PRE)
Source: PLoS One. 2014 Apr 17;9(4):e93561. doi: 10.1371/journal.pone.0093561 (PMC3990577; doi:10.1371/journal.pone.0093561)
Supplement: Supplementary Information S1 — Isolation of chromatin from Drosophila larvae. Section 1 describes protocol to isolate Drosophila Larvae chromatin. Section 2 describes protocol to perform Chromatin Immunoprecipitation on Drosophila Larvae. (DOC) [file pone.0093561.s006.doc]

***Isolation of chromatin from Drosophila larvae***

Briefly, 150-200 mg of third instar larvae were homogenised in 5ml of buffer A1(60 mM KCl, 15 mM NaCl, 4 mM MgCl_2_, 15 mM HEPES pH 7.6, 0.5% Triton X100, 0.5 mM DTT, 10 mM sodium butyrate and protease inhibitor [Roche Protease Inhibitor Cocktail tablet]) containing 1.8% Formaldehyde using Dounce homogeniser. The homogenate was incubated for 15 minutes at room temperature following which 540 µl of 2.5 M solution of glycine was added and further incubation was carried out at room temperature for 5 minutes. The homogenate was centrifuged for 5 minutes at 1500 g. The pellet was washed 3 times with buffer A1 and the pellet was resuspended in 0.5 ml of lysis buffer(140 mM NaCl, 1 mM HEPES pH 7.6, 1 mM EDTA, 0.5 mM EGTA, 1% Triton X-100, 0.5 mM DTT, 0.1% sodium deoxycholate, 0.05% SDS, 10 mM sodium butyrate and protease inhibitors). The mixture was incubated on an agitator for 10 minutes at 4°C. The extracted chromatin was sonicated in a Biorupter (Diagenode) for 12.5 min at high setting and 30 sec on/off cycle to obtain DNA fragments 200-1000 bp in size.

***Chromatin immunoprecipitation***

For each sample, 20 µg of chromatin pre-cleared with 40 µl Protein A agarose beads (Millipore) was taken and diluted with 1.8 ml of ChIP dilution buffer (16.7 mM Tris Cl. pH 8.1, 1.2 mM EDTA pH 8.0, 167 mM NaCl, 0.05% SDS, 1.1% Triton X-100). 2 µg of appropriate antibody was added for each set of chromatin Immunoprecipitation. The antibody was allowed to bind to the chromatin overnight on an agitator at 4°C followed by addition of 60 µl protein A- agarose beads and agitation at 4°C for further 2 hours. The beads with bound chromatin fraction was centrifuged and washed with low salt wash buffer(20 mM Tris Cl. pH 8.0, 2 mM EDTA pH 8.0, 150 mM NaCl, 0.05% SDS, 1% Triton X-100), high salt wash buffer(20 mM Tris Cl. pH 8.0, 2 mM EDTA pH 8.0, 500 mM NaCl), LiCl wash buffer(10 mM Tris Cl. pH 8.1, 1 mM EDTA pH 8.0, 0.25 M LiCl_2_, 1% NP40, 1% deoxycholic acid) and twice with TE buffer. The chromatin agarose bead complex was then incubated in 500 µl of elution buffer (1% SDS, 0.1 M NaHCO_3_) at room temperature for 20 minutes. For reverse cross linking between proteins and DNA 20µl of 5 M NaCl was added to the supernatant reverse cross and incubated at 65°C for 4 hours. DNA was isolated by Proteinase K digestion followed by Phenol /Chloroform extraction and precipitated using isopropanol and 20 µg glycogen as carrier. The isolated DNA was stored in 20 µl RNase / DNase free water.
